# KSHV Reprogramming of Host Energy Metabolism for Pathogenesis

**DOI:** 10.3389/fcimb.2021.621156

**Published:** 2021-05-12

**Authors:** Xiaoqing Liu, Caixia Zhu, Yuyan Wang, Fang Wei, Qiliang Cai

**Affiliations:** ^1^ Ministry of Education (MOE) & National Health Committee (NHC) & Chinese Academy of Medical Science (CAMS), Key Laboratory of Medical Molecular Virology, Department of Medical Microbiology and Parasitology, School of Basic Medicine, Shanghai Medical College, Fudan University, Shanghai, China; ^2^ Sheng Yushou Center of Cell Biology and Immunology, School of Life Science and Biotechnology, Shanghai Jiao Tong University, Shanghai, China

**Keywords:** KSHV, energy metabolism, viral reprogramming, oncogenesis, herpesvirus

## Abstract

Reprogramming of energy metabolism is a key for cancer development. Kaposi’s sarcoma-associated herpesvirus (KSHV), a human oncogenic herpesvirus, is tightly associated with several human malignancies by infecting B-lymphocyte or endothelial cells. Cancer cell energy metabolism is mainly dominated by three pathways of central carbon metabolism, including aerobic glycolysis, glutaminolysis, and fatty acid synthesis. Increasing evidence has shown that KSHV infection can alter central carbon metabolic pathways to produce biomass for viral replication, as well as the survival and proliferation of infected cells. In this review, we summarize recent studies exploring how KSHV manipulates host cell metabolism to promote viral pathogenesis, which provides the potential therapeutic targets and strategies for KSHV-associated cancers.

## Introduction

**Figure 1:**
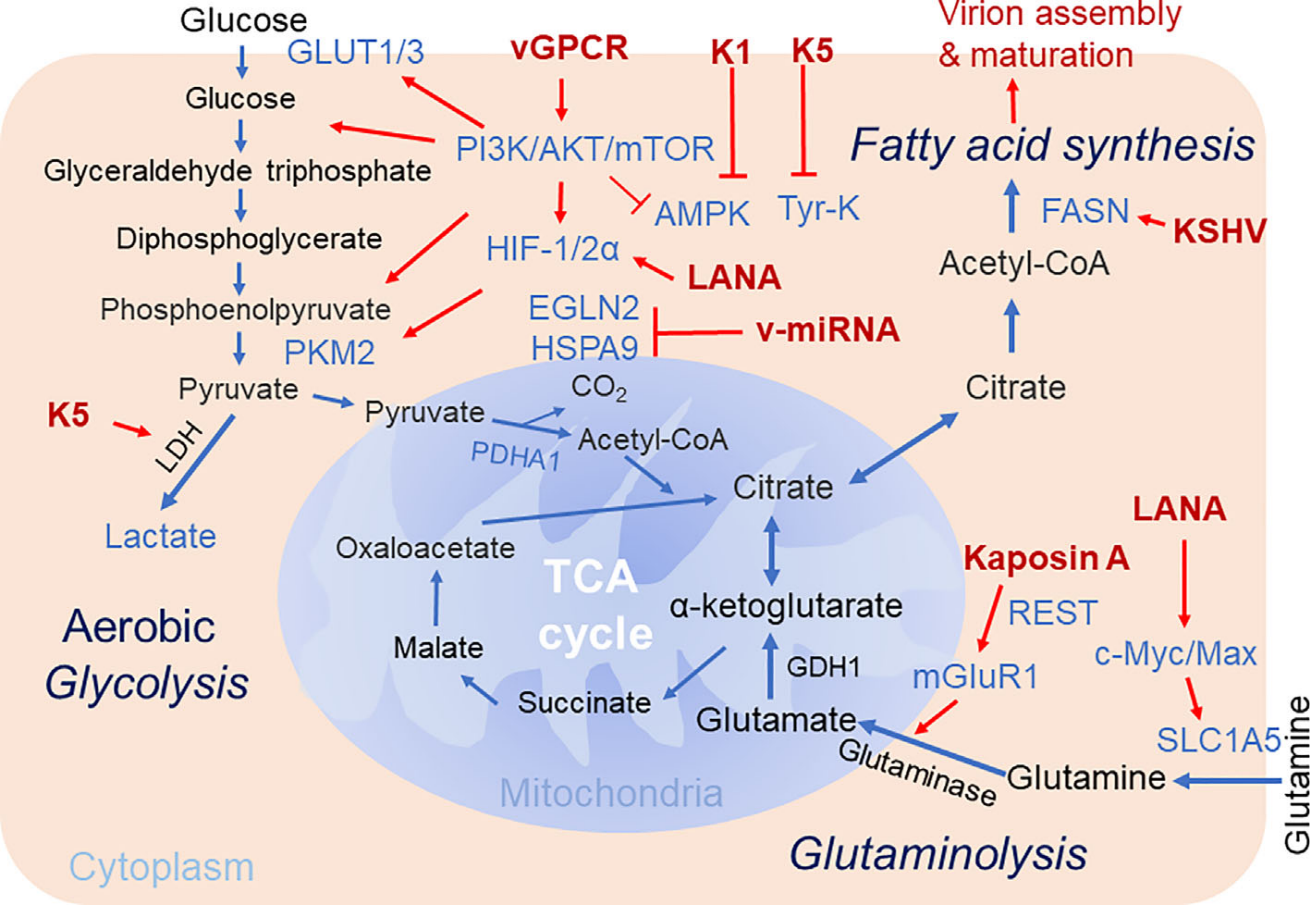
Brief schematics of KSHV-mediated alteration of host cellular metabolic pathways. The key steps of three cellular metabolic pathways including aerobic glycolysis, glutaminolysis and fatty acid synthesis targeted by KSHV for pathogenesis are highlighted. The increased expression of enzymes and metabolites are shown in blue. The KSHV-encoded proteins are shown in red. Red arrow denotes the regulation by KSHV. TCA Cycle, tricarboxylic acid cycle; GDH, glutamate dehydrogenase; PKM2, pyruvate kinase 2; Tyr-K, Tyrosine kinase; EGLN2, HIF prolyl hydroxylase 2; mGluR1, metabotropic glutamate receptor 1; SLC1A5, glutamine transporter.

Reprogramming of energy metabolism is a hallmark of cancers. Cancer cells often rewire metabolic pathways to balance ATP production for supporting rapidly tumor proliferation. In normal cells, glucose is converted into pyruvate through glycolysis, and pyruvate is oxidized in mitochondria to produce ATP (**Figure 1**). While in cancer cells, pyruvate is preferentially catabolized into lactate even in the presence of oxygen, this metabolic process is known as “aerobic glycolysis” or “Warburg effect” (Delgado et al., 2010; Courtnay et al., 2015). In Warburg effect, glucose-derived carbon is directed away from the TCA cycle and ATP production is decreased. For producing energy sufficient to maintain cancer cell proliferation, glutaminolysis is also often elevated in most of cancer cells. Glutaminolysis is shown to replenish the TCA cycle *via* converting glutamine to glutamate, which is in turn transaminated to α-ketoglutarate and produces citrate within mitochondria, while a proportion of this citrate is exported into the cytoplasm, and cytoplasmic citrate is then metabolized into acetyl-CoA for fatty acid synthesis and related biosynthetic precursors (Biswas et al., 2012; Dyer et al., 2019). This process is also termed as anaplerosis, and is a dominant metabolism mode in rapidly growing malignant cells besides Warburg effect. Thus, the metabolic plasticity of cancer cells is essential to generate energy required for cell proliferation.

Increasing evidence has shown that virus infection, similar to cancer development, depends on the reprogramming of cellular metabolism to produce biomass for viral replication and virion production. Virus infection may reprogram host metabolism for viral genome and protein synthesis, as well as lipid envelope generation for virion production. Interestingly, in the oncoviruses-associated cancers, accumulating literatures have also reported that many oncogenic viruses alter the function of pivotal cellular proteins that involve in reprogramming of cell metabolism to facilitate cancer development in addition to supporting virus propagation (Yu et al., 2018; Purdy and Luftig, 2019).

**Figure 2:**
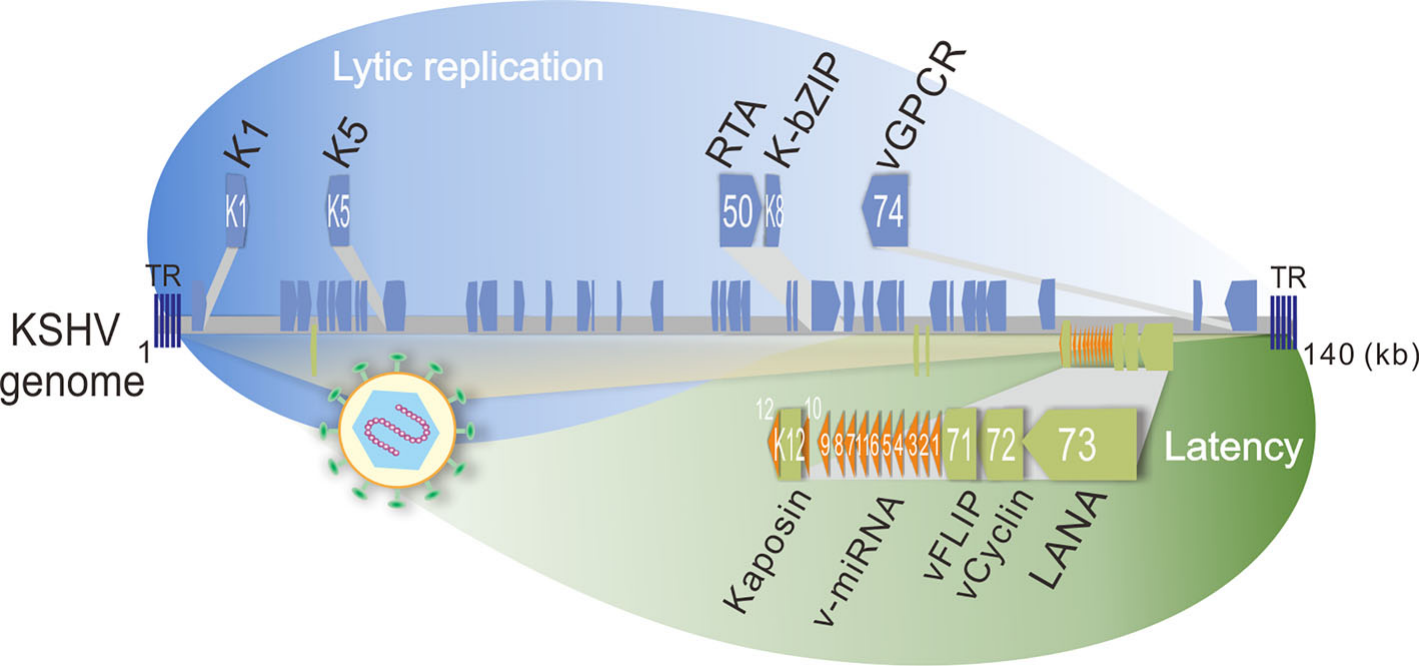
Schematic representation of KSHV-encoded proteins that involve in host energy metabolism reprogramming during latency and lytic replication. The lytic genes (K1, K5, orf50, K8, and orf74 which encodes K1, K5, RTA, K-bZIP, and vGPCR, respectively) are shown on the top panel, and the latent genes (K12, v-miRNA, orf71, orf72, and orf73 which encodes Kaposin, v-miRNA cluster, vFLIP, vCyclin, and LANA, respectively) are shown at the bottom panel. TR, terminal repeat; kb, kilo base pair.

Kaposi’s sarcoma-associated herpesvirus (KSHV), a human oncogenic gamma-herpesvirus, can establish life-long latent infection in human. KSHV is tightly associated with Kaposi’s sarcoma (KS) and two B-cell lymphomas including primary effusion lymphoma (PEL) and multicentric Castleman disease (MCD) by infecting B-lymphocyte and endothelial cells, respectively. Like other herpesviruses, KSHV contains two phases of life cycle: latency and lytic replication (**Figure 2**). During latency, only a limited number of proteins including LANA (Latency-Associated Nuclear Antigen, ORF73), vCyclin (viral Cyclin, ORF72), vFLIP (viral FLIP, ORF71), Kaposin (K12), and 12 microRNAs (v-miRNA) are expressed, which play critical roles in host cell transformation and growth. In contrast, the majority of the KSHV genome-encoded genes including K1, K5, K-bZIP (K8), and vGPCR (viral G Protein-Coupled Receptor, ORF74) are activated with the expression of the lytic master regulator of RTA (ORF50) during lytic replication. Within the tumor tissue, most of the infected cells are in latency state, only about 5% of the cells undergo lytic replication (Staskus et al., 1997). It has been assumed that both latent and lytic replicative cycles of KSHV contribute to tumor formation and development (Ganem, 2006). Since viruses do not possess a metabolic network but require metabolites for viral survival, it becomes a barrier for virus replication and inducing tumorigenesis. To overcome this barrier, it has been demonstrated that KSHV has evolved multiple strategies to alter metabolic pathways, including aerobic glycolysis, fatty acid synthesis, oxidative phosphorylation, reactive oxygen species (ROS) production, glutaminolysis and amino acid synthesis, which appears to participate in virus production at different stages of the viral life cycle (Delgado et al., 2012; Sanchez et al., 2017). In addition, KSHV also alters the metabolic pathways for sustaining and promoting the proliferation and survival of host cells, particularly under stress conditions (Singh et al., 2019; Münz, 2020). Thus, the metabolic properties of KSHV-infected cells are closely resembled to the metabolic hallmarks of cancer cells.

Given both viral replication and tumorigenesis are energy-consuming processes, it is not surprising that the regulation of host cell metabolism is a barrier for establishment of oncovirus persistent infection and invasion. Considering there are multiple similarities between metabolic reprogramming by oncogenic viruses and cancer cells, the alterations in cancer-associated metabolic reprogramming may play profound effects on viral gene expression, cell transformation, and the response to tumor microenvironment during the life cycle of viral infection (Pavlova and Thompson, 2016). In this review, we summarize most recent studies regarding to how KSHV alters host cellular metabolism for viral production and oncogenesis, particularly three major pathways of cellular metabolism including central carbon metabolism, aerobic glycolysis, glutaminolysis and fatty acid synthesis are addressed and discussed below (**Figure 1**).

## KSHV Infection Alters the Host Central Carbon Metabolism

It has been shown that KSHV latent infection can induce aerobic glycolysis and lactic acid production with decrease of oxygen consumption, which is consistent with the Warburg effect (Delgado et al., 2010). Inhibitors of glycolysis, including Oxamate and 2-Deoxyglucose, is able to selectively induce apoptosis of the KSHV-infected endothelial cells instead of mock cells (Delgado et al., 2010), indicating that KSHV-transformed endothelial cells depend on glycolysis to establish viral latency infection and survival.

In addition, it has also been demonstrated that KSHV latently-infected endothelial cells are prone to take advantage of glutamine, and glutamine deprivation or glutamine transporter inhibition could induce cell death of KSHV latently-infected cells (Sanchez et al., 2015). Unlike most cancer cells that primarily utilize glutamine and asparagine to complete the TCA cycle, KSHV-infected cells depend on glutamine and asparagine to provide γ-nitrogen for purine and pyrimidine biosynthesis (Zhu et al., 2017). Knockdown of any one of three rate-limiting enzymes in the *de novo* purine biosynthesis pathway, including phosphoribosyl pyrophosphate aminotransferase (PPAT), phosphoribosyl pyrophosphate synthetases 1 and 2 (PRPS1/2), or a rate-limiting enzyme like carbamoylphosphate synthetase 2 (CAD) in the pyrimidine biosynthesis pathway, will dramatically impair the proliferation of KSHV-latently transformed endothelial cells instead of its untransformed cells (Zhu et al., 2017). This indicates that the glutamine-dependent nucleotide biosynthesis is required for the anabolic proliferation of KSHV-infected cells.

A global metabolomics analysis of KSHV-infected cells indicates that metabolites of several pathways, including glycolysis, amino acid metabolism, the pentose phosphate pathway and lipogenesis, are commonly deregulated by KSHV. For example, KSHV infection elevates majority of the detectable metabolite products of *de novo* fatty acid synthesis (FAS), and inhibition of the key enzymes in fatty acid synthesis will lead to apoptosis of KSHV-infected cells but not uninfected cells (Delgado et al., 2012), suggesting that increased level of fatty acid synthesis is also essential for survival of KSHV latently-infected endothelial cells. In addition to endothelial cells, KSHV also induces B-cell lymphoma, including B-cell non-Hodgkin lymphomas (B-NHL) and primary effusion lymphoma (PEL), to maintain high levels of glycolysis and fatty acid synthesis. It has been revealed that aerobic glycolysis and FAS occur in a PI3K-dependent manner, and the fatty acid synthesizing enzyme (FASN) is overexpressed in PEL (Sommermann et al., 2011; Bhatt et al., 2012).

In cancer cells, the induction of glycolysis and fatty acid synthesis is considered to serve as a resource for rapid energy production, or as other specific metabolites necessary for increased cell division or proliferation (DeBerardinis et al., 2008). In addition to deregulation of multiple cellular metabolic pathways during KSHV latent infection, emerging evidence shows that KSHV also modifies host cellular carbon source utilization to ensure optimal environments for viral DNA replication and virion production during lytic replication. For example, Sanchez et al. demonstrated that KSHV lytic replication depends on the host major central carbon metabolic pathways. Inhibition of glycolysis, glutaminolysis, or fatty acid synthesis could efficiently reduce KSHV virion production from both endothelial lytic system and the iSLK cell-inducible KSHV system (Sanchez et al., 2017).

## Mechanisms by Which KSHV Controls Aerobic Glycolysis, Glutaminolysis and Fatty Acid Synthesis

A better understanding of how KSHV switches to cancer-like cell metabolism will be helpful in seeking novel therapies for KSHV infection. Increasing evidence shows that KSHV reprograms host cell metabolism mainly through targeting directly regulation of metabolic enzymes at the transcriptional or translational levels, or indirectly activating metabolic regulators that are commonly occurred in cancer cells, such as HIF-1α, c-MYC, AMPK and so on, to alter host metabolic gene expression (**Table 1**).

**Table 1:** List of KSHV-encoded proteins reprogramming host metabolism.

Viral gene	Target pathway	Mechanism of metabolism rewiring	Ref
LANA	Aerobic glycolysis	Stabilization of HIF-1α by degrading the E3 ubiquitin ligase VHL, the stabilized HIF-1α upregulates PKM2	(Cai et al., 2006; Ma et al., 2015)
K1	Aerobic glycolysis	Inactivation of AMPK	(Anders et al., 2016)
vGPCR	Aerobic glycolysis	Increased expression of HIF-1α and HIF-2α in a mTOR-dependent manner	(Jham et al., 2011)
K5	Aerobic glycolysis	Modulating endocytosis of cellular growth factor-binding receptor-associated tyrosine kinase	(Karki et al., 2011)
v-miRNAs	Aerobic glycolysis	Down-regulation of EGLN2 and HSPA9 to decrease mitochondrial biogenesis	(Yogev et al., 2014)
LANA	Glutaminolysis	Upregulation of the glutaminase and glutamate by activating c-Myc	(Valiya Veettil et al., 2014)
Kaposin A	Glutaminolysis	Increased expression of the metabotropic glutamate receptor 1	(Valiya Veettil et al., 2014)

### Aerobic Glycolysis

It has been demonstrated that KSHV-encoded microRNAs (v-miRNA) within the latent gene cluster collaborate to induce aerobic glycolysis with decreased oxygen consumption, increased lactate secretion and glucose uptake. Mechanistically, v-miRNA cluster alters host cell energy metabolism through regulating the translation and mRNA stability of their target genes, such as HIF prolyl hydroxylases 2 (EGLN2) and the mitochondrial heat shock protein A9 (HSPA9). Knockdown of any one of these gene, will stabilized HIF1α, decreased oxygen consumption and reduced mitochondria volume (Yogev et al., 2014). It was recently shown that the KSHV v-miRNAs can be transferred to the surrounding uninfected cells to induce a reverse Warburg effect by exosomes. ‘Reverse Warburg Effect’ is a new term that means cancer cells can induce aerobic glycolysis in adjacent stromal cells except for their intrinsic metabolic alteration. This raises the possibility that viruses could use exosomes to shape the metabolism of host tissue microenvironment during persistent infection (Yogev et al., 2017). In addition, some lytic gene products can also contribute to transform endothelial cells. For examples, KSHV-encoded K5, as the first discovery of viral E3 ubiquitin ligase, is able to increase aerobic glycolysis and lactate production, as well as promote oncogenesis through modulating endocytosis of host cellular growth factor-binding receptor-associated tyrosine kinase, which in turn increases the sensitivity of cells to autocrine and paracrine factors (Karki et al., 2011).

Hypoxia-inducible factor-1α (HIF-1α) is an intracellular key factor that mediates many cellular responses to hypoxia. Intensive studies have demonstrated that HIF-1α is a metabolic regulator which regulates the transcription of genes involved in aerobic glycolysis or fatty acid synthesis during cancer development and oncovirus infection (Singh et al., 2016; Lo et al., 2017). It has been shown that KSHV targets and activates HIF-1α, and several KSHV-encoded genes are in turn activated by HIF-1α, suggesting HIF-1α might play a substantial role in KSHV-induced oncogenesis. Previous studies have shown that LANA encoded by KSHV stabilize HIF-1α by degrading the E3 ubiquitin ligase VHL in the KSHV latently-infected B cells (Cai et al., 2006), and the stabilized HIF-1α in turn upregulates pyruvate kinase 2 (PKM2) (which is the key step enzyme of the glycolytic to maintain aerobic glycolysis in infected cells) and increases lactate production (Ma et al., 2015). Consistence with this observation, HIF-1α knockdown in PEL cell lines results in a reduction of both aerobic/anaerobic glycolysis and lipid biogenesis, indicating that HIF-1α is necessary for maintaining a metabolic state optimal for growth of PEL (Shrestha et al., 2017). Interestingly, some studies have also revealed that the expression of the lytic oncoprotein vGPCR leads to induction of both HIF-1α and HIF-2α in a mTOR-dependent manner (Jham et al., 2011). Given that PI3K-AKT-mTOR pathway is essential for controlling cell proliferation and regulating anabolic activities in B lymphocytes and endothelial cells (Tomlinson and Damania, 2004; Wang et al., 2004), PI3K/AKT signaling is also shown to involve in regulation of aerobic glycolysis by controlling the expression and localization of the glucose transporter 1/3 (GLUT1/3) and other glycolytic enzymes (Manning and Cantley, 2007), or inactivating the negative regulator of aerobic glycolysis AMPK (Wang and Damania, 2008). For example, the KSHV-encoded K1 protein has been shown to inactivate AMPK for deregulation of aerobic glycolysis by binding to its γ- subunit (Anders et al., 2016).

### Glutaminolysis

Glutamine is the other major substrate that contributes to energy production. It has been shown that glutamine is usually transported into cells through glutamine transporter SLC1A5, and then converted into a-ketoglutarate at the catalysis of glutamate dehydrogenase 1 (GDH1) to replenish TCA cycle (Daye and Wellen, 2012), or shunt a-ketoglutarate into citrate for lipid production, which is called gxlutaminolysis. The profile analysis of high-throughput RNA sequencing has revealed that the expression levels of many enzymes (such as glutaminase 2, GLS2; glutamate dehydrogenase 1, GDH1; and glutamic-oxaloacetic transaminase 2, GOT2) in the glutamine pathway are upregulated by KSHV infection, which supports the notion that KSHV mediates host cellular glutaminolysis for promoting proliferation of KSHV-transformed cells (Sanchez et al., 2015; Zhu et al., 2017).

During latent infection, it is not only KSHV-encoded LANA protein upregulates the glutaminase and glutamate by activating c-Myc, the latent viral protein Kaposin A also upregulates expression of the metabotropic glutamate receptor 1 (mGluR1 receptor) by binding and sequestering host REST (RE-1 Silencing Transcription Factor) in the cell cytoplasm to relieve the REST-mediated suppression of the mGluR1 gene. Binding of glutamate to mGluR1 in turn induces signaling and proliferation of infected cells (Valiya Veettil et al., 2014). In addition, abundant evidence has demonstrated that extended c-Myc network contributes to glutamine addiction in some cancers (Dang, 2013). Consistence with the discovery that MondoA/Mlx signaling pathway is essential for regulation of glutaminolysis in cancer cells, KSHV also induces expression of the heterodimeric transcription factors c-Myc-Max and their related heterodimer MondoA-Mlx to upregulate glutamine transporter SLC1A5, leading to increased glutamine uptake for glutaminolysis in the KSHV-infected endothelial cells (Sanchez et al., 2015).

### Fatty Acid Synthesis

Fatty acid, as the main component of all biological lipid membranes, is another essential substrate for energy metabolism, and appears to play a role in cancer pathogenesis (Dang, 2013). Although the result from metabolomics analysis showing that an increase in fatty acid precursor metabolites is generally associated with increased FAS (i. e. choline and phosphocholine), however, the metabolites of glycerophosphorylcholine and glycerol-3-phosphate, that are associated with fatty acid production, are tend to be reduced due to degradation of phospholipids (Delgado et al., 2012). Despite whether KSHV activates fatty acid synthesis remains undefined, it is still possible that KSHV increases the fatty acid synthesis-related enzymes to promote its synthesis. In addition, the increased acetyl-CoA from glutaminolysis flux may be the material for *de novo* synthesis of fatty acids.

Similar to the observation that KSHV-transformed cells depend on glutamine for their growth, proliferation, and survival (Zhu et al., 2017), the induction of fatty acid synthesis is required for the survival of KSHV latently-infected endothelial cells, and inhibition of FAS will greatly increase apoptotic death of the latently infected cells instead of uninfected counterparts (Sanchez et al., 2017). Therefore, the product of FAS is necessary for the survival of endothelial cells with KSHV latent infection.

Although both glycolysis and glutaminolysis have been shown to involve in viral genome replication, they are also required for regulation of lytic gene expression at different early steps. For examples, glycolysis is necessary for early gene transcription, while glutaminolysis is necessary for early gene translation. Likewise, inhibition of fatty acid synthesis leads to a reduction of KSHV infectious particles production without defects in viral gene expression or replication (Sanchez et al., 2017). Similar phenomenon is also observed that inhibition of FAS results in reduction of infectious particles in other enveloped viruses including EBV (Li et al., 2004), HCMV, influenza virus (Munger et al., 2008), varicella-zoster virus (VZV) and HCV (Yang et al., 2008), supporting the notion that FAS is essential for the viral envelop membrane synthesis (Delgado et al., 2012), albeit the precise mechanism remains to be further investigated. Therefore, inhibition of these specific cellular metabolic pathways could not only eliminate latently infected cells but also block lytic replication (Sanchez et al., 2017), which will provide the potential therapeutic targets and strategies against KSHV-associated cancers.

## Conclusion and Prospective

In this review, we focus to address how KSHV infection rewires host cellular metabolism. During KSHV infection, aerobic glycolysis, glutaminolysis and fatty acid synthesis are the major pathways for host cell energy mass production, while other pathways including amino acid metabolism and the pentose phosphate pathway are also altered. EBV, another gamma-herpesvirus with high homologues of KSHV, has also been shown to manipulate many metabolic pathways by encoding different viral proteins (Piccaluga et al., 2018), particularly a recent study showing that EBV can remodel mitochondrial one-carbon (1C) metabolism to drive B-cell transformation (Wang et al., 2019). This indicates that KSHV may also rewire other pathways by encoding various viral proteins, in addition to only few of them related to metabolic pathways are reported so far. It can also be concluded that KSHV alters host cells metabolism mainly through the activation of viral oncogenes or the common host metabolic regulators such as HIF-1α and c-Myc, which is similar as that has been demonstrated in the EBV-transformed B-cell lymphomas (Piccaluga et al., 2018). Thus, the studies about how KSHV infection alters host metabolism for the survival of latently infected cells will provide novel therapeutic strategies for eliminating latent infection of KSHV, and will also shed a light on how a virus targets host cell metabolism for driving tumor development.

Although intensive studies have focused to address how metabolic reprogramming influences the propagation and survival of host cells, how metabolic alteration influences virus replication or production is still unclear. Further studies are required for uncover how metabolic reprogramming impacts the viral life cycles, particularly KSHV. Due to both immune response and immune escape play crucial roles in viral infection, whether metabolic reprogramming involves in those processes is still an open question and need be further investigation in future. Uncovering metabolic factors that contribute to anti-virus or viral infection will also provide novel therapeutic targets against oncoviruses infection and their associated cancers.

## Author Contributions

XL drafted the manuscript. CZ and YW provided critical reading. FW and QC supervised final manuscript. All authors contributed to the article and approved the submitted version.

## Funding

This work was supported by the National Natural Science Foundation of China (81971930, 81772166), the National Key Research and Development Program of China (2019ZX09721001), and the Research Program on Biosafety Guarantee Technology of High-level Biosafety Laboratory and Important Pathogen Laboratory (2018ZX10734401-004).

## Conflict of Interest

The authors declare that the research was conducted in the absence of any commercial or financial relationships that could be construed as a potential conflict of interest.
